# First case report of hypouricemia associated with adjuvant imatinib therapy in a patient with small intestinal gastrointestinal stromal tumor

**DOI:** 10.3389/fonc.2026.1765401

**Published:** 2026-05-20

**Authors:** Juan Bu, Yeledan Mahan, Reziwanguli Yuemaier, Xiaoling Zhang, Ling Zhou

**Affiliations:** Department of Medical Research and Translational Management, People’s Hospital of Xinjiang Uygur Autonomous Region, Urumqi, China

**Keywords:** adverse event, GIST, hypouricemia, imatinib, tyrosine kinase inhibitor

## Abstract

Imatinib, a tyrosine kinase inhibitor, is primarily used for the treatment of chronic myeloid leukemia and gastrointestinal stromal tumors (GIST). The most common adverse events include peripheral edema, mild nausea, vomiting, diarrhea, myalgia, muscle cramps, and skin rash. In patients with GIST receiving imatinib therapy, routine laboratory tests typically reveal hematologic abnormalities such as decreased hemoglobin, erythrocyte, neutrophil, and platelet counts, alongside elevated hepatic transaminase levels. However, to our knowledge, no cases of imatinib-associated hypouricemia have been reported. Here, we present a case of hypouricemia temporally associated with adjuvant imatinib therapy in a patient with intestinal GIST. This case highlights the importance for clinicians to be aware of hypouricemia as a potential adverse event during imatinib therapy.

## Introduction

Gastrointestinal stromal tumor (GIST), the most common soft tissue sarcoma of the gastrointestinal tract, accounts for 3% of gastric cancers (1). The pathogenesis of GIST is primarily associated with mutations in KIT or platelet-derived growth factor receptor alpha (PDGFRA) (2). For patients with resectable non-metastatic GIST, surgical resection remains the standard first-line treatment. However, for unresectable, metastatic, or recurrent GISTs, tyrosine kinase inhibitors (TKIs) currently represent the mainstay of therapy (3).

Imatinib mesylate (Gleevec), a selective inhibitor of receptor tyrosine kinases, was first approved in 2001 for the treatment of chronic myeloid leukemia (CML) and subsequently for patients with KIT-positive unresectable and/or metastatic malignant GIST, as well as for adjuvant therapy in adult patients with KIT-positive GIST at significant risk of recurrence after surgical resection (4, 5). Long-term imatinib therapy has been shown to significantly improve prognosis and overall survival of patients with GIST (6).

Common adverse events (AEs) include peripheral edema, mild nausea, vomiting, diarrhea, myalgia, muscle cramps, and skin rash. Severe events, although rare, may lead to irreversible organ damage or life-threatening complications. Hematologic manifestations typically involve decreased hemoglobin, red blood cell counts, neutrophil, and platelets, along with elevated alanine aminotransferase (ALT) and aspartate aminotransferase (AST). Hyperuricemia has been sporadically documented in the literatures, but there have been no reports to date, of imatinib-associated hypouricemia. Here, we present a novel case of hypouricemia observed during adjuvant imatinib therapy for intestinal GIST.

## Case presentation

A 48-year-old Han Chinese woman was incidentally found to have an abdominal solid mass during routine health screening on November 15, 2020. Initial laboratory tests revealed normal liver and renal function, a serum uric acid (SUA) level of 163 μmol/L (reference range: 155–357 μmol/L), and an unremarkable complete blood count.

On December 24, 2020, the patient was diagnosed with small intestinal GIST. Following exclusion of surgical contraindications, an R0 resection was successfully performed on December 31, 2020. Histopathological examination confirmed a moderate-risk GIST (tumor diameter: 5 cm, mitotic index: 2/50 HPF) (7). Immunohistochemical staining demonstrated positive expression of CD117, DOG1, CD34, Ki-67 (5%), and SDHB. Comprehensive genomic profiling of the tumor tissue (15 GIST-related genes analyzed including whole-exome sequencing for 11 genes, targeted hotspot testing for pharmacogenomic variants in 1 gene, and rearrangement analysis for 3 genes) identified *KIT* exon 11 insertion-deletion (indel) mutation without other genetic abnormalities.

Postoperatively, a liquid diet was commenced on day three, and a regular diet was resumed within two weeks, with no evidence of malnutrition. Adjuvant imatinib mesylate (Gleevec, Novartis) therapy was initiated on February 28, 2021, at a daily dosage of 400 mg with dinner. The patient had no other comorbidities or concomitant medications. Liver and renal function remained normal throughout postoperative follow-up.

Follow-up laboratory monitoring revealed progressive hematologic abnormalities commencing with isolated erythrocytopenia in April 2021, developing into persistent decreases in erythrocytes, hemoglobin, and leukocytes from August 2021 onward. These abnormalities were monitored but did not require intervention following clinical assessment. SUA levels during treatment showed a declining trend compared with the pre-treatment baseline of 163 μmol/L: 113.3 μmol/L (April 6, 2021), 108 μmol/L (August 27, 2021), 120 μmol/L (December 28, 2021), 144 μmol/L (August 11, 2023), and 96 μmol/L (November 14, 2024). It demonstrated a persistent downward trend relative to the patient’s own reference level.

**Figure 1:**
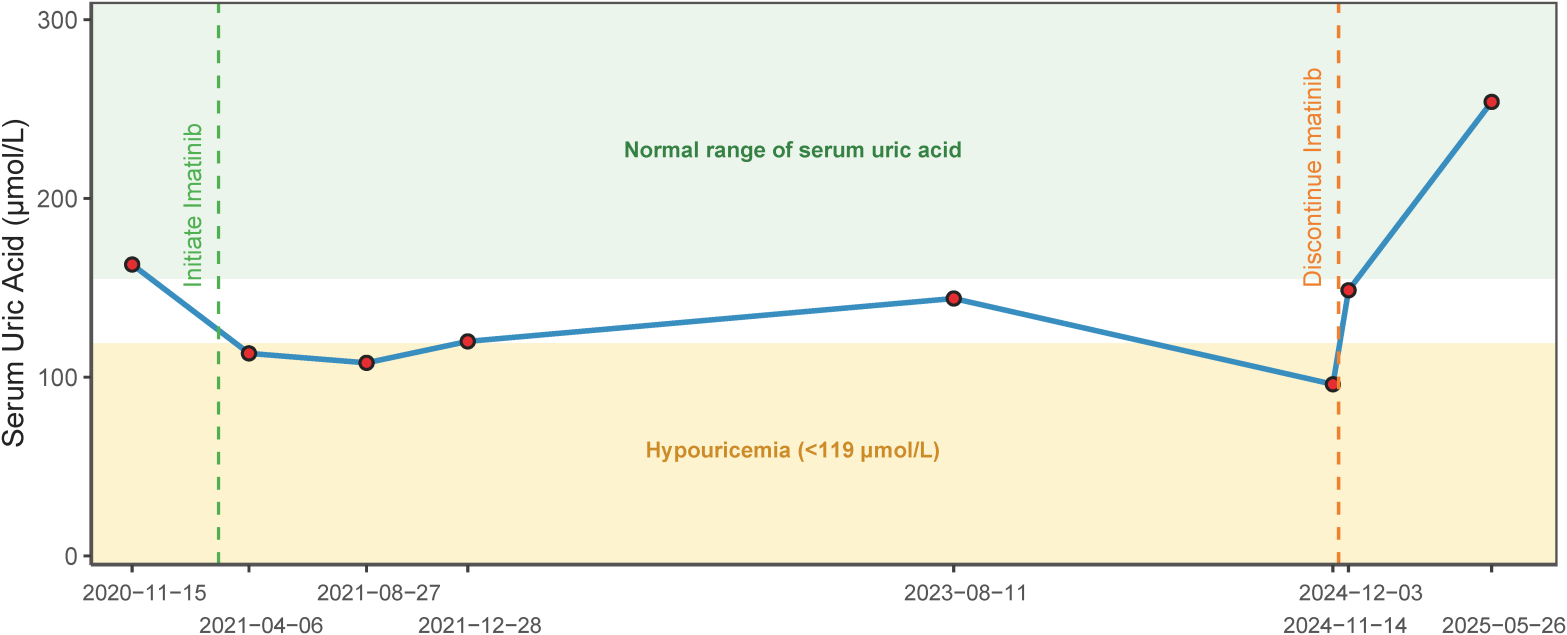
Temporal correlation between serum uric acid levels and imatinib administration.

Health checkup on November 14, 2024 identified rectal polyps during a surveillance colonoscopy, with normal abdominal imaging. In preparation for polypectomy, the imatinib dosage was adjusted to 400 mg every other day starting November 14, 2024, and discontinued one week later (November 21, 2024). Polypectomy was performed on December 3, 2024, and histology subsequently confirmed the polyps were benign. Concurrent laboratory tests showed SUA nearly normalized (148.6 μmol/L), though hematologic abnormalities persisted. By May 26, 2025, both SUA (254 μmol/L) and other hematologic parameters had fully normalized. The temporal trend of SUA levels is shown in **Figure 1**.

## Discussion

Gastrointestinal stromal tumors (GISTs) are mesenchymal tumors originating from the interstitial cells of Cajal or their stem cell precursors, most commonly arising in the stomach and small intestine (8). Early-stage patients are usually asymptomatic, and the tumors are often detected incidentally. With disease progression, symptoms such as nausea and vomiting, abdominal pain and distension, or gastrointestinal bleeding may develop. In this case, the patient had neither relevant family history nor allergies, and the GIST was discovered during a routine examination. Immunohistochemical analysis showed positive expression of CD117, DOG-1, and CD34. The tumor was in the small intestine with a maximum diameter of 5 cm. Genetic analysis revealed an indel mutation in exon 11 of *KIT*. Considering the tumor size, location, and the sensitivity of *KIT* exon 11 mutation to imatinib, adjuvant imatinib therapy was recommended. The patient began oral administration of imatinib at 400 mg daily one month after surgery.

Studies have confirmed that adjuvant imatinib is generally well-tolerated by patients with GIST. Saito et al. found that among imatinib-treated GIST patients, grade 3 or 4 AEs occurred in neutropenia (10%), anemia (15%), and renal impairment (5%) (9). Martin et al. reported that frequent AEs were fluid retention, nausea, fatigue, rash, gastrointestinal discomfort, and myalgia, with anemia being the predominant laboratory abnormality (10). In a randomized, phase III, double-blind, placebo-controlled, multicenter trial, DeMatteo et al. observed that grade 1 or 2 AEs were common and mostly involving gastrointestinal effects, headache, rash, edema, fatigue, or myalgias/arthralgias (11). In a study of 129 Chinese GIST patients, Zhang et al. found that the most common AEs associated with imatinib were edema (51.94%), followed by leukopenia (40.31%) and rash (24.81%). Rare AEs such as interstitial pneumonia (1.55%), alopecia (1.55%), and neurological dysfunction (dizziness [1.55%], insomnia [1.55%], headache [0.78%]) were also reported (12). Notably, Cheng et al. reported hyperuricemia (16.9%) in a long-term follow-up study of imatinib-treated chronic-phase CML patients (13). In the present case, liver and renal function remained normal throughout imatinib treatment. While decreases in erythrocytes, hemoglobin, and leukocytes were noted, these changes did not require intervention following clinical assessment. The patient also experienced facial edema, nausea, and abdominal pain. These symptoms were mild and persisted throughout the imatinib therapy, without an obvious temporal relationship to SUA levels. Remarkably, we observed a persistent decline in SUA levels following imatinib initiation, with the lowest recorded SUA level at 96 μmol/L. After discontinuation of imatinib in preparation for polypectomy, the patient’s SUA gradually returned to normal. This temporal reversibility supports a probable association between imatinib therapy and hypouricemia. To our knowledge, this represents the first reported instance of hypouricemia associated with imatinib. No such reports exist in the literature on imatinib’s AEs, nor for other TKIs used in GIST treatment. To quantify this association, the Naranjo Adverse Drug Reaction Probability Scale (14) was applied, yielding a score of 6, indicating a probable association (**Table 1**). This assessment strengthens the evidence for imatinib as a likely contributing factor, particularly given the absence of other hypouricemia-inducing factors and the temporal correlation with treatment initiation and discontinuation.

**Table 1:** Adverse drug reaction probability scale by Naranjo.

Assessment	Yes	No	Unknown	Score
1. Are there previous conclusive reports on this reaction?	+1	0	0	0
2. Did adverse event appear after the suspected drug was given?	+2	−1	0	+2
3. Did the adverse reaction improve when the drug was discontinued or a specific antagonist was given?	+1	0	0	+1
4. Did the adverse reaction appear when the drug was readministered?	+2	−1	0	0
5. Are there alternative causes that could have caused the reaction?	−1	+2	0	+2
6. Did the reaction reappear when a placebo was given?	−1	+1	0	0
7. Was the drug detected in any body fluid in toxic concentrations?	+1	0	0	0
8. Was the reaction more severe when the dose was increased, or less severe when the dose was decreased?	+1	0	0	0
9. Did the patient have a similar reaction to the same or similar drugs in any previous exposure?	+1	0	0	0
10. Was the adverse event confirmed by any objective evidence?	+1	0	0	+1

Uric acid plays an important role in physiological and pathological processes. Increasing evidence indicates a U-shaped association between SUA levels and all-cause mortality. Hypouricemia, defined as SUA ≤ 119 μmol/L, has drawn growing clinical attention. Zou et al. found a 4.7-fold increased prevalence of cardiovascular diseases among hypouricemic rheumatoid arthritis (RA) patients compared to normouricemic RA patients (15). A cross-sectional study by Gao et al. using U.S. NHANES data (1999–2020) revealed that both low and high SUA levels increased stroke risk in different populations (16). Other studies have linked hypouricemia to increased risk of diabetes in Chinese (Wang et al.) and hypertension in Japanese (Kawasoe et al.) (17, 18). Furthermore, hypouricemia is also implicated in the pathogenesis of neurodegenerative disorders such as Parkinson’s disease, dementia, and multiple sclerosis. Hypouricemia is associated with multiple adverse health outcomes, underscoring the importance of clinician awareness regarding the potential harm of low SUA levels in patients.

Uric acid is primarily a product of purine metabolism *in vivo*, and hypouricemia may result from either decreased production or increased renal excretion. The main causes include malnutrition, renal tubular dysfunction, liver dysfunction, mutations in urate transporter genes such as URAT1, GLUT9, GLUT12, and XOR, as well as drug effects (**Table 2**). In the present case, no etiological factor other than imatinib use was identified. Macioszek et al. utilized a GIST xenograft mouse model comprising 10 imatinib-treated and 10 untreated control mice and performed GC-MS metabolomic analysis of tumor extracts (19). They found that purine metabolic components including hypoxanthine, xanthine, xanthosine, and uric acid were significantly reduced. In cancer, purine demand is increased. If purine synthesis is insufficient, tumor cells cannot proliferate. Therefore, imatinib’s anticancer activity may be partly mediated through inhibition of purine biosynthesis. However, there is currently no direct evidence that imatinib reduces uric acid production through inhibition of purine synthesis, and the mechanism by which imatinib may cause hypouricemia requires further investigation. To date, reported AEs of TKIs have predominantly involved hyperuricemia. TKIs, including ruxolitinib, regorafenib, and sunitinib, have been associated with elevated uric acid levels and hyperuricemia, which is generally attributed to tumor lysis syndrome (20, 21). Since a reduction in uric acid is commonly regarded as a clinical benefit rather than an AE, this may explain why hypouricemia associated with TKIs has not been previously reported. Nevertheless, excessively low SUA levels may increase the risk of various diseases. Therefore, in clinical practice, prolonged markedly low SUA (≤180 μmol/L) should be avoided where possible, and drug-induced hypouricemia should be promptly reported as an adverse drug reaction, in order to support updates to prescribing information and promote rational clinical drug use.

**Table 2:** Most commonly used medications that may cause hypouricemia.

Major	Category	Subcategory	Drug(s)	Possible mechanisms	References
Uric Acid–Lowering Therapies (Used for Hyperuricemia/Gout)	Xanthine Oxidase (XO) Inhibitors	purine analogue	Allopurinol	Inhibit XO, reducing UA production Possible stimulation of MRP4-mediated UA transport May regulate OAT1, OAT3, and URAT1 expression, increasing UA excretion	(22–25)
		non-purine	Febuxostat	Inhibit both oxidase and dehydrogenase forms of XO, reducing UA production Inhibit ABCGs, reducing restoration of UA	(22, 26)
	Uricosuric Agents		Probenecid, Benzbromarone	Restrain UA reabsorption by URAT1 Related to OAT1, OAT3, OAT4 and OAT10, promoting renal excretion of UA Inhibit MRP4-mediated UA transport (Probenecid has biphasic effect)	(24, 27–29)
	Urate Oxidases		Pegloticase, Rasburicase	Convert UA to allantoin (more soluble), thus reducing serum UA	(30, 31)
Drugs Used for Other Purposes but Indirectly Lower Uric Acid	Antihypertensives	Angiotensin II Receptor Blockers (ARBs)	Losartan	Reduce UA crystals by urine alkalinization Inhibit URAT1, MRP4, OAT1, OAT3 and OAT10, increasing UA excretion	(27, 32)
		Angiotensin-Converting Enzyme (ACE) Inhibitors	Captopril, Ramipril, Enalapril	Inhibit URAT1 and OAT4 (Captopril), reducing reabsorption of UA	(29, 33, 34)
		Calcium Antagonists	Amlodipine, Felodipine, Nifedipine, etc	Inhibit renal UA reabsorption by URAT1 Increase renal blood flow and GFR; reduce UA precursor production	(35–37)
	Lipid Lowering Drugs	Fibrates	Fenofibrate	Inhibit URAT1, OAT3 and ABCG2	(27, 38)
		Statins	Atorvastatin, Simvastatin	Increase renal blood flow Inhibit OAT3	(35)
	Antidiabetics	SGLT2 Inhibitors	Empagliflozin, Dapagliflozin	Increase UA excretion through GLUT9 pathway Promote ABCGs expression; regulate URAT1	(39, 40)
	Anti-inflammatory Drugs	NSAIDs	high dose aspirin (>3g/day)	Inhibit URAT1	(28)

UA, uric acid; MRP4, multi-drug resistance-associated protein 4; OAT, organic anion transporter; URAT1, urate anion transporter 1; ABCGs, ATP-binding cassette transporter; G2, *GFR* glomerular filtration rate; *SGLT2*, sodium glucose cotransporter 2; GLUT9, glucose transporter 9; NSAIDs, non-steroidal anti-inflammatory drug.

## Conclusion

In this case, the patient’s SUA declined progressively during adjuvant imatinib therapy, reaching a nadir of 96 μmol/L, which met the diagnostic threshold for hypouricemia. To our knowledge, there is no reported case of hypouricemia as a potential AE of imatinib. While causality cannot be definitively established from a single case, the temporal relationship between imatinib initiation and SUA decline, along with its normalization following drug discontinuation and a Naranjo score of 6, collectively support a probable association. These findings suggest that clinicians may consider monitoring SUA levels in imatinib-treated patients and further studies are warranted to confirm and elucidate this potential AE.

## Data Availability

The original contributions presented in the study are included in the article/**Supplementary Material**. Further inquiries can be directed to the corresponding author.
