# Supplementary material for: First case report of hypouricemia associated with adjuvant imatinib therapy in a patient with small intestinal gastrointestinal stromal tumor
Source: Front Oncol. 2026 May 20;16:1765401. doi: 10.3389/fonc.2026.1765401 (PMC13229782; doi:10.3389/fonc.2026.1765401)
Supplement: Supplementary file 1 [file DataSheet1.zip › original test data/Pathology report.pdf]

[redacted]医院

病理检查补充报告单

[redacted]

病理号：[redacted]

姓　名：[redacted]　　年　龄：45 岁　　性 别：　女

民　族：汉族　　送检单位：本院　　联系电话：[redacted]

住院号：[redacted]　　床　号：17　　送检医生：[redacted]

送检日期：2021-01-04　　送检科室：胃肠外科病区

大体描述：

（小肠）部分小肠，长5.5cm，一侧断端周径为5cm，另一侧断端周径为4.3cm，距5cm断端2.6cm，距另一侧断端2.2cm，黏膜略平坦，黏膜下见一肿物，大小为5×3×3cm，肿物切实质中，切面灰红，其余小肠粘膜灰黄，皱壁明显。

病理图像：

Color Original Image Downloaded from EHR

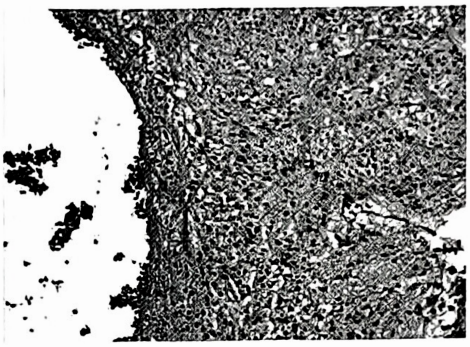

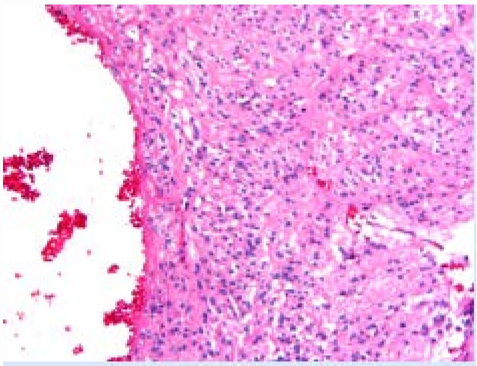

病理诊断：

（部分小肠）胃肠道间质瘤，肿瘤最大径约5cm，核分裂像约2个/5平方毫米，属于低危险度。
免疫组化结果：SMA(-)，Desmin(-)，CD117(+)，DOG-1(+)，CD34(+)，S-100(-)，Ki-67(+)5%，SDHB(+)。

报告医生：[redacted]　　**审核医生：**[redacted]　　**诊断日期：** 2021-01-08

电话：[redacted]　邮编：83001　地址：[redacted]
