# Supplementary material for: First case report of hypouricemia associated with adjuvant imatinib therapy in a patient with small intestinal gastrointestinal stromal tumor
Source: Front Oncol. 2026 May 20;16:1765401. doi: 10.3389/fonc.2026.1765401 (PMC13229782; doi:10.3389/fonc.2026.1765401)
Supplement: Supplementary file 1 [file DataSheet1.zip › original test data/sequencing report.pdf]

1、基本信息

姓名：[redacted]	送检医院：[redacted]	订单编号：[redacted]
性别：女	送检科室：-	样本编号：[redacted]
年龄：44岁	送检医生：-	取材部位：-
联系电话：[redacted]	门诊/住院号：-	标本类型：石蜡切片
临床诊断：胃肠道间质瘤	采样时间：2021.01.09	接收时间：2021.01.11

2、检测项目

检测范围及内容：

检测项目	检测内容	检测意义
体细胞变异检测	15个基因：11个基因全部外显子，1个基因用药相关的热点区域，3个基因重排分析<br>突变形式：点突变、小片段插入缺失、拷贝数变异以及融合	预测靶向药物（FDA/NMPA批准药物、指南推荐以及临床试验药物）的药敏性

检测平台：Illumina（NextSeq 550AR/NovaSeq 6000）

参考基因组：GRCh37/hg19

3、关键信息摘要

检出变异情况	靶向用药相关基因变异	KIT p.I563_P577delinsT

靶向用药提示*	FDA/NMPA批准、NCCN/CSCO指南建议本癌种可能获益药物	瑞戈非尼; 伊马替尼
	FDA/NMPA批准、NCCN/CSCO指南建议其他癌种可能获益药物	伊马替尼
	临床研究阶段潜在获益药物	pexidartinib＋PLX9486; PLX9486; 舒尼替尼; 索拉非尼; 达沙替尼; Ponatinib; 尼洛替尼
	临床前研究阶段潜在获益药物	Avapritinib

*注：可能耐药的药物：标注红色的为NCCN指南提示可能耐药的药物，黑色的为较低证据支持（尚未达成专家共识）的可能耐药药物，如选择此类药物需密切注意疗效。

其他药物推荐

药物	证据来源
Cabozantinib	胃肠道间质瘤,NCCN,2021.V1，推荐Cabozantinib用于治疗批准疗法失败后的患者。
尼洛替尼<br>Nilotinib	胃肠道间质瘤,NCCN,2021.V1，推荐尼洛替尼用于治疗批准疗法失败后的患者。
培唑帕尼<br>Pazopanib	胃肠道间质瘤,NCCN,2021.V1，推荐培唑帕尼用于治疗批准疗法失败后的患者。
Ripretinib	FDA：用于治疗已接受3种或更多种激酶抑制剂（包括伊马替尼）治疗的晚期胃肠道间质瘤（GIST）成人患者。
瑞戈非尼<br>Regorafenib	FDA：适用于既往接受过甲磺酸伊马替尼及苹果酸舒尼替尼治疗的局部晚期的、无法手术切除的或转移性的胃肠道间质瘤患者。<br>NMPA：既往接受过甲磺酸伊马替尼及苹果酸舒尼替尼治疗的局部晚期的、无法手术切除的或转移性的胃肠道间质瘤（GIST）患者。<br>胃肠道间质瘤,NCCN,2021.V1，推荐依维莫司+瑞戈非尼用于治疗批准疗法失败后的患者。
舒尼替尼<br>Sunitinib	FDA：用于治疗使用伊马替尼甲磺酸盐后疾病进展或不耐受的胃肠道间质瘤患者。<br>NMPA：甲磺酸伊马替尼治疗失败或不能耐受的胃肠间质瘤（GIST）。<br>胃肠道间质瘤,NCCN,2021.V1，推荐依维莫司+舒尼替尼用于治疗批准疗法失败后的患者。
索拉非尼<br>Sorafenib	胃肠道间质瘤,NCCN,2021.V1，推荐索拉非尼用于治疗批准疗法失败后的患者。
伊马替尼<br>Imatinib	胃肠道间质瘤,NCCN,2021.V1，推荐依维莫司+伊马替尼用于治疗批准疗法失败后的患者。<br>NMPA：用于治疗不能切除和/或发生转移的恶性胃肠道间质瘤（GIST）的成人患者。

4、基因变异结果汇总

4.1 靶向用药相关基因变异

基因变异	突变丰度/拷贝数	变异分级	可能敏感药物		可能耐药药物
			本癌种	其他癌种	
KIT<br>NM_000222.2<br>exon11<br>p.I563_P577delinsT<br>c.1688_1729delTAAATGGAAACAATTATGTTTACATAGACCCAACACAACTTC	33.27%	I类	瑞戈非尼（A级）<br>伊马替尼（A级）<br>pexidartinib<br>+PLX9486 (C级)<br>PLX9486 (C级)<br>Ponatinib (C级)<br>达沙替尼（C级）<br>尼洛替尼（C级）<br>舒尼替尼（C级）<br>索拉非尼（C级）<br>Avapritinib (E级)	伊马替尼（C级）	无

注：

1. 基因突变："exon"为外显子，"intron"为内含子，"c"为DNA序列，"p"为蛋白质，"NM"为基因的转录本编号。
   常见突变类型展示及说明如下：
   （1）错义突变：如L858R，表示第858位氨基酸由L突变为R（其中L为野生型氨基酸，R为突变型氨基酸）；
   （2）无义突变：如Q171*，表示第171位氨基酸由Q突变为终止密码子，*代表编码终止；
   （3）移码突变：如p.Y225Ffs*17，表示第225位氨基酸由Y突变为F，并继续编码16个氨基酸后终止；
   （4）整码突变：如c.2235_2249del，p.E746_A750del表示在746-750位置氨基酸发生缺失；
   （5）剪切突变：如c.36+1G>T，表示c.36前面一个外显子的最后一个碱基位于编码区36位，+1代表这个外显子挨着的后面的内含子的第一个核苷酸发生突变，碱基由G突变为T；
   （6）融合突变：如EML4(E13)-ALK(E20)融合，表示EML4基因第13外显子和ALK基因第20外显子发生融合；
   （7）拷贝数变异：如拷贝数扩增/缺失，表示基因发生了拷贝数扩增/缺失变异。
2. 突变丰度：在某位点产生突变的等位基因在该位点全部等位基因中所占比率。例如，突变丰度10%意为该位点含有10%的突变等位基因和90% 的野生型等位基因。基因拷贝数：是指某一种基因或某一段特定的DNA序列在单倍体基因组中出现的数目。
3. 根据AMP/ASCO/CAP 共识《Standards and Guidelines for the Interpretation and Reporting of Sequence Variants in Cancer》结合《临床分子病理实验室二代基因测序检测专家共识》：
   （1）基因变异与治疗、诊断、预后的证据级别共分为五个等级：A 级（FDA或NMPA批准，或来自于NCCN/CSCO指南用于本癌种的治疗方案），B 级（尚未进入诊疗指南，来自领域专家共识的较大规模的临床研究），C 级（FDA或NMPA批准，或来自于NCCN/CSCO指南用于其他癌种的治疗方案、或者已作为临床试验的筛选入组标准、或者有多个小型研究支持），D 级（病例研究），E 级（临床前研究）。
   （2）基因变异按照临床意义的重要性分为四个类别：I类：具有明确临床意义的变异（A级或B级证据）；II类：具有潜在临床意义的变异（C级、D级或E级证据）；III类：临床意义不确定的变异（尚无相关临床证据）；IV 类：无害或可能无害的变异（在全人群或特定人群数据库中观察到高变异频率），报告中未列出。
4. 报告所呈现的变异命名参考HGVS的命名规则（http://www.hgvs.org/mutnomen/）。
5. 受样本取材或探针设计等因素影响或限制，NGS在某些变异类型如拷贝数扩增的检测方面有时存在一定的局限性。对于NGS方法准确性可能有影响的情况，应考虑采用可靠的单基因方法对检出变异进行验证。

4.2 临床意义尚不明确的基因变异

基因变异	突变丰度/拷贝数	变异分级	蛋白功能影响	功能预测		数据库编号 (COSMIC)
				SIFT	Polyphen2	
未检出						

4.3胃肠道间质瘤诊疗相关基因结果汇总

检测基因	覆盖区域	检测结果
BRAF	全部外显子	未见突变
FGFR1	全部外显子	未见突变
KIT	全部外显子	第9号外显子未见突变<br>**第11号外显子插入缺失突变**<br>第12号外显子未见突变<br>第13号外显子未见突变<br>第14号外显子未见突变<br>第17号外显子未见突变<br>第18号外显子未见突变<br>其他外显子未见突变
KRAS	全部外显子	未见突变
NF1	第1-26外显子；第28-58外显子	未见突变
NRAS	全部外显子	未见突变
NTRK1	第8-17外显子；第9-12内含子	未见突变
NTRK2	第12-20外显子；第15内含子	未见突变
NTRK3	第12-20外显子；第13-14部分内含子	未见突变
PDGFRA	全部外显子	第12号外显子未见突变<br>第14号外显子未见突变<br>第18号外显子未见突变<br>其他外显子未见突变
PIK3CA	全部外显子	未见突变
SDHA	全部外显子	未见突变
SDHB	全部外显子	未见突变
SDHC	全部外显子	未见突变
SDHD	全部外显子	未见突变

5、样本质控

质量参数	评估参数	实际结果	质控标准
DNA质量评估	DNA提取总量	7033.6ng	≥15ng
	DNA片段降解程度	合格	合格
	DNA质量总评估	合格	合格
文库质量评估	文库总量	1598.0ng	≥300ng
	文库质量总评估	合格	合格
测序质量评估	平均测序深度	1468.9X	≥1200 X
	Q30比率	93.7%	≥80%
	测序质量总评估	合格	合格
参考样本质量评估	阳性参考品	合格	合格
	阴性参考品	合格	合格
	参考样本质量总评估	合格	合格
	总体质量评估	合格	

注：

1.中度溶血样本、重度溶血样本、黄疸样本可能存在大片段基因组DNA污染，存在假阴性可能；中度脂血、重度脂血样本中可能存在抑制PCR扩增的干扰物质，可能影响最终结果的准确性。

2.DNA提取总量：送检样本DNA提取总量。

3.DNA片段降解程度：利用片段分析仪器评估DNA片段的降解程度。

4.DNA质量总评估：通过DNA总量、降解程度综合评估样本的质量。

5.文库总量：将原始DNA加接头后经扩增纯化得到的中间产物总量。

6.平均测序深度：目标基因每个碱基被测到的平均次数。

7.Q30比率：是指错误识别的概率是0.1%，即错误率0.1%，或者正确率是99.9%。

8.测序质量总评估：结合平均测序深度和Q30比率综合评估测序质量。

9.阳性参考品：在已知位点发生了突变的标准参考品，在同一实验体系中进行检测，预期这些位点都能检出为阳性结果，符合预期则代表该实验体系质控达标。

10.阴性性参考品：在已知位点未发生突变的标准参考品，在同一实验体系中进行检测，预期这些位点检出为阴性结果，符合预期则代表该实验体系质控达标。

11.参考样本质量总评估: 结合阴阳性参考品综合评估参考样本质量。

12.总体质量评估：综合DNA质量总评估、测序质量总评估以及血液样本性状进行综合评估样本质量，分为”合格”、”警戒”、”不合格”三个等级，质控 ”警戒”、”不合格” 可能会影响检测的准确性和灵敏度。

13.NA：样本不适用或未进行此项目评估。

14.质控只针对肿瘤DNA样本进行评估。

组织病理质控结果

样本HE染色镜下图片

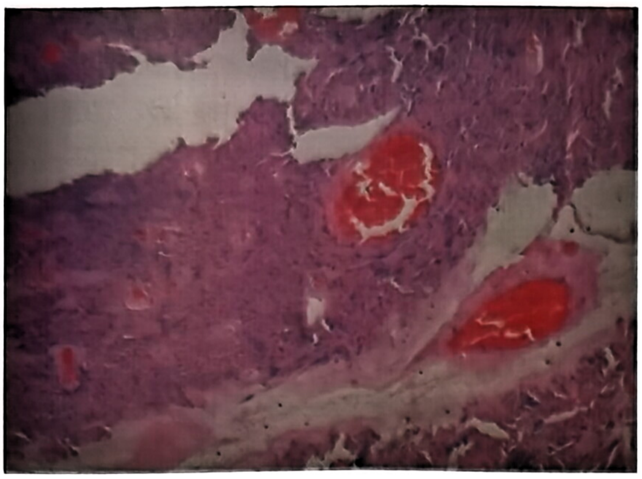
210102953-1

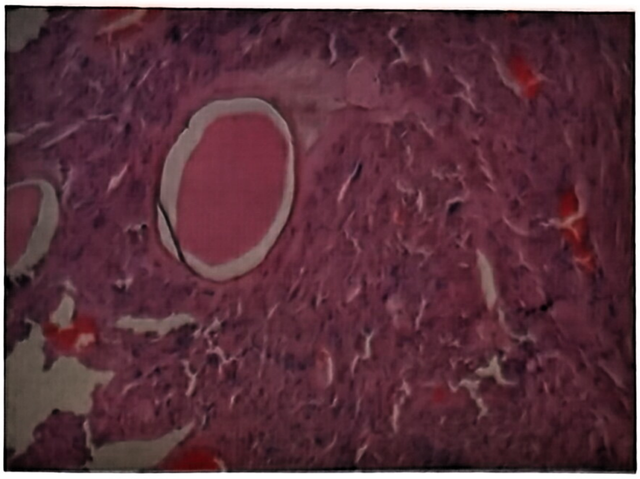
210102953-2

样本评估结果

肿瘤细胞占比	肿瘤面积	坏死细胞占比	是否圈片提取
90%	约 120mm^2	0%	否

组织病理评估情况
**合格**

注:

1.本报告只对该样本进行组织病理评估，评估结果包括肿瘤细胞占比，肿瘤面积，坏死细胞占比。

2.本报告只对符合病理评估条件的样本进行出具，如送样没送白片则无此报告。

3.本报告对送检的其中一张白片进行病理评估，其结果仅做参考。

4.该评估结果不适用于肿瘤良恶及肿瘤性质的判断，仅作为对下一步检测样本肿瘤细胞占比的分析前质量评估。

5.组织病理评估结果分为 ”合格”、”警戒”、”不合格”三个等级。是根据肿瘤细胞占比多少、肿瘤面积多小、坏死细胞占比多少进行的综合评估结果。

6.为提升肿瘤细胞检出率，本实验室对可圈片样本进行圈片提取，该部分样本肿瘤细胞占比仅作参考。
