# Supplementary figures and images for: First case report of hypouricemia associated with adjuvant imatinib therapy in a patient with small intestinal gastrointestinal stromal tumor
Source: Front Oncol. 2026 May 20;16:1765401. doi: 10.3389/fonc.2026.1765401 (PMC13229782; doi:10.3389/fonc.2026.1765401)

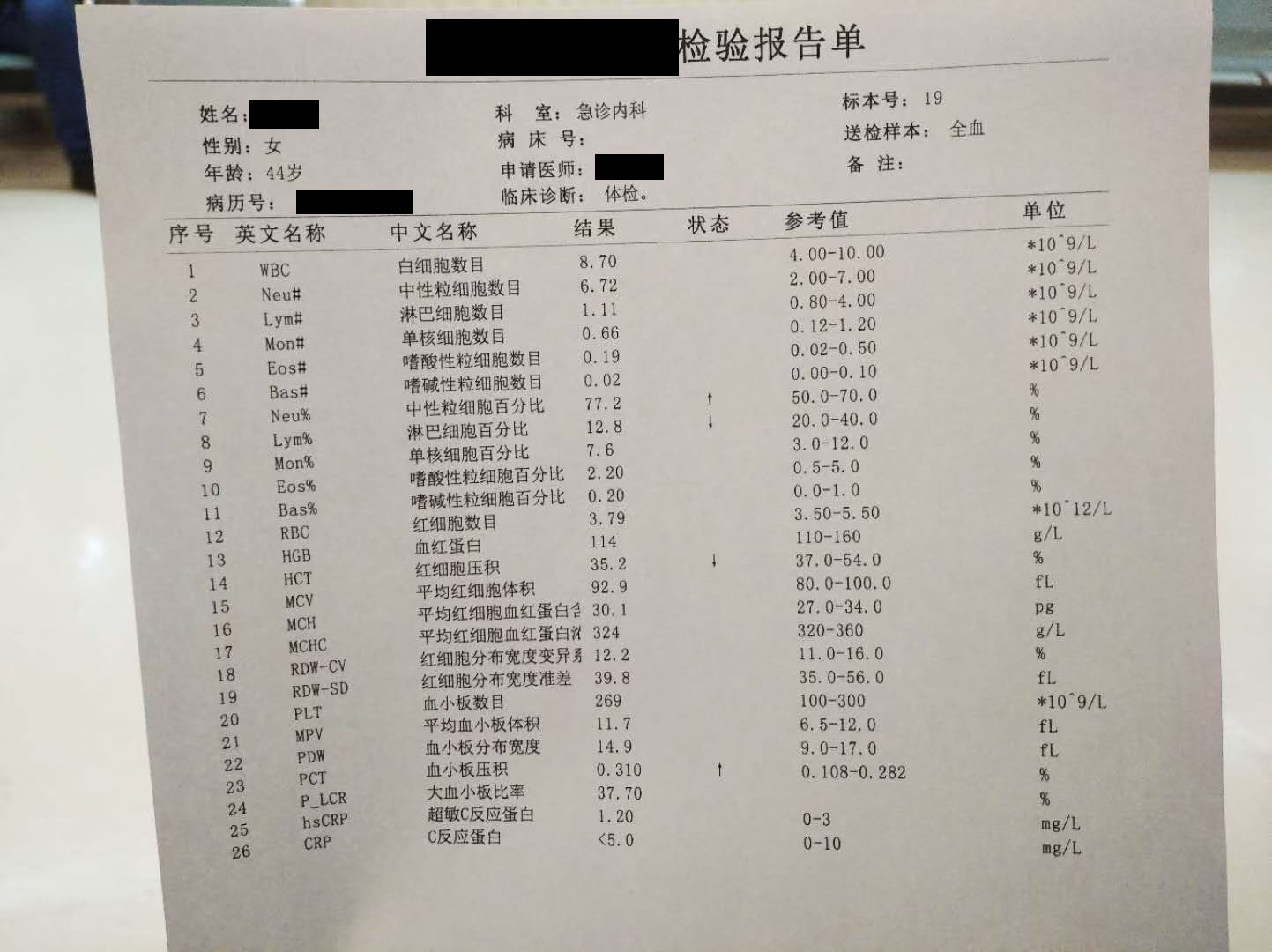

Supplement: Supplementary file 1 [file DataSheet1.zip › original test data/2020.11.15—CBC.jpg]

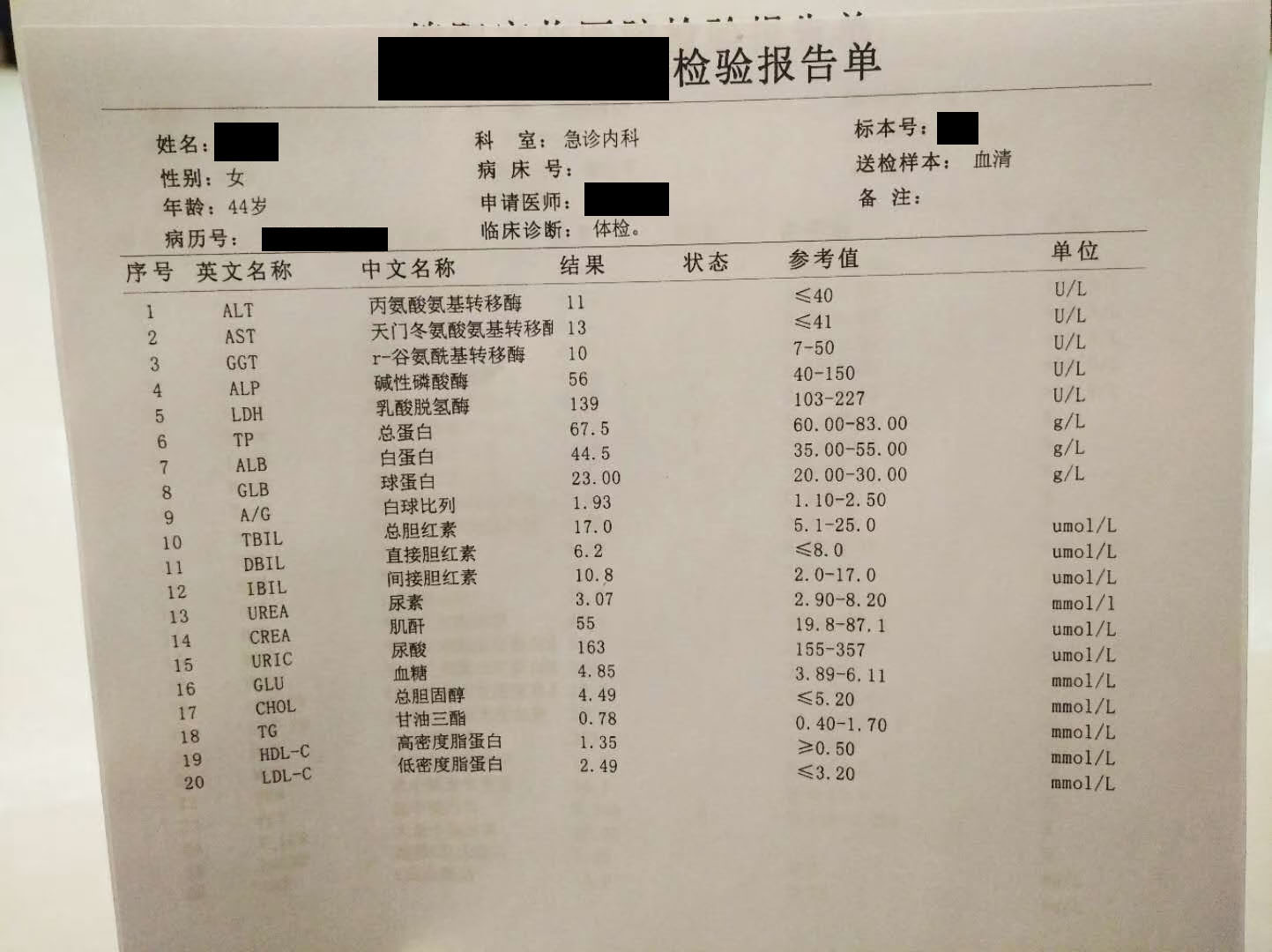

Supplement: Supplementary file 1 [file DataSheet1.zip › original test data/2020.11.15—CMP.jpg]

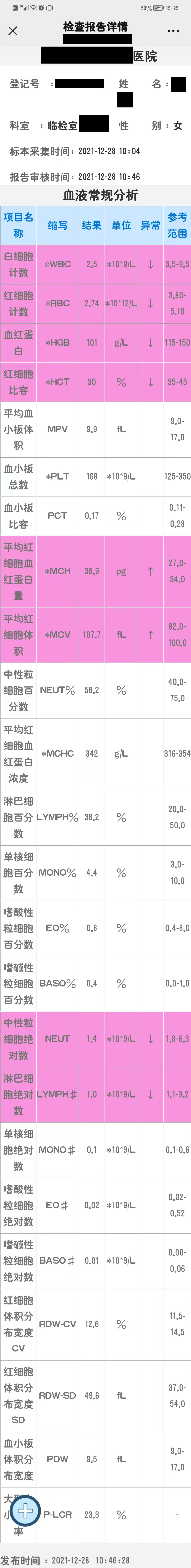

Supplement: Supplementary file 1 [file DataSheet1.zip › original test data/2021.12.28—CBC.jpg]

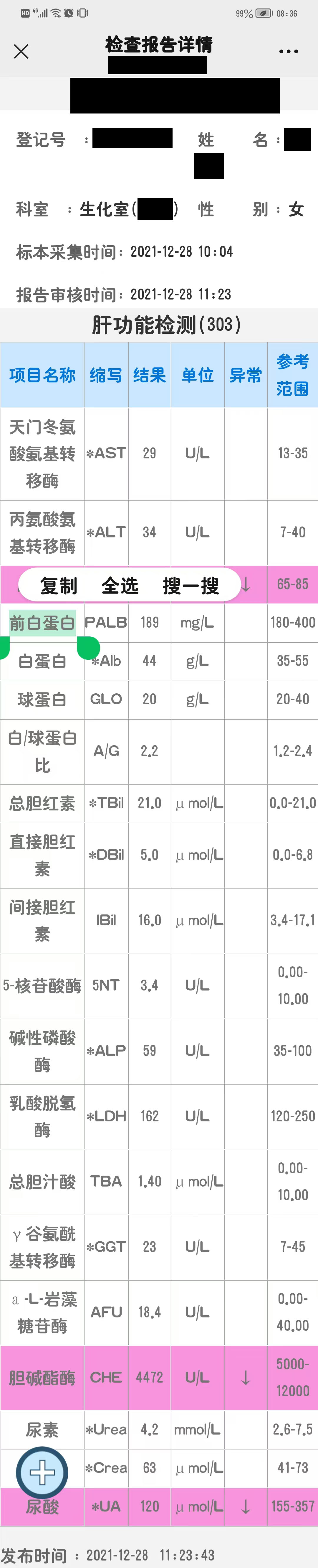

Supplement: Supplementary file 1 [file DataSheet1.zip › original test data/2021.12.28—CMP.jpg]

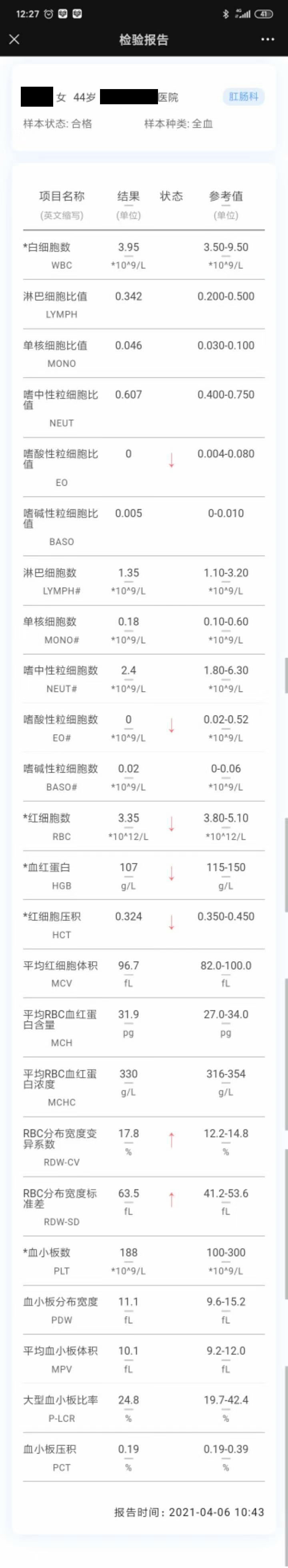

Supplement: Supplementary file 1 [file DataSheet1.zip › original test data/2021.4.6—CBC.jpg]

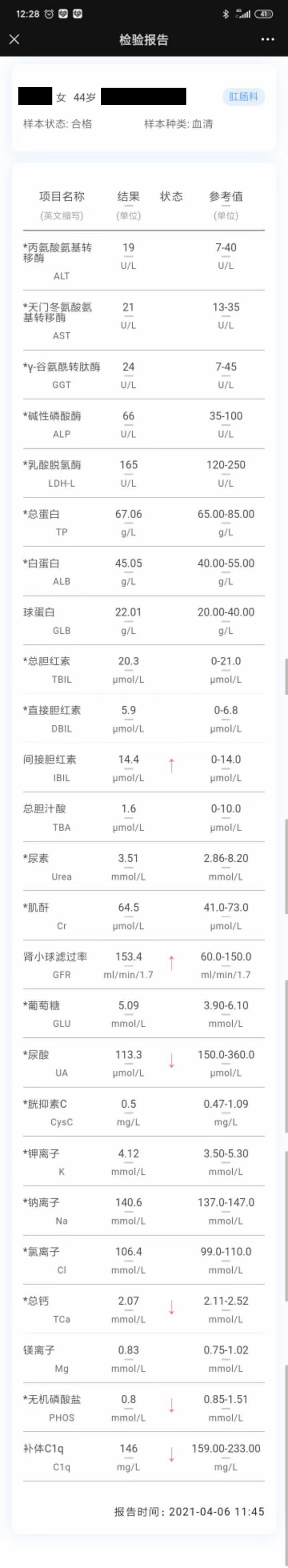

Supplement: Supplementary file 1 [file DataSheet1.zip › original test data/2021.4.6—CMP.jpg]

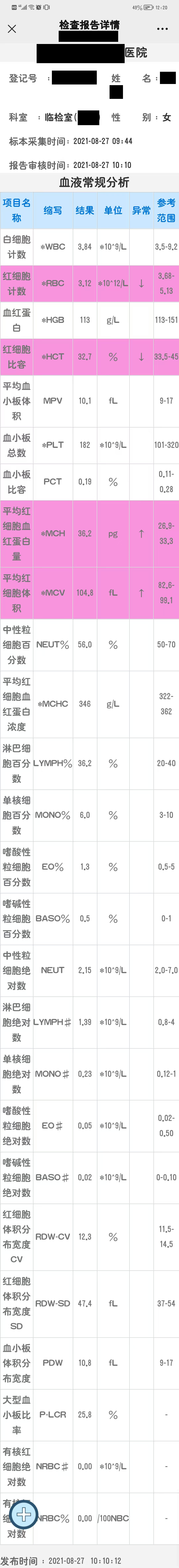

Supplement: Supplementary file 1 [file DataSheet1.zip › original test data/2021.8.27—CBC.jpg]

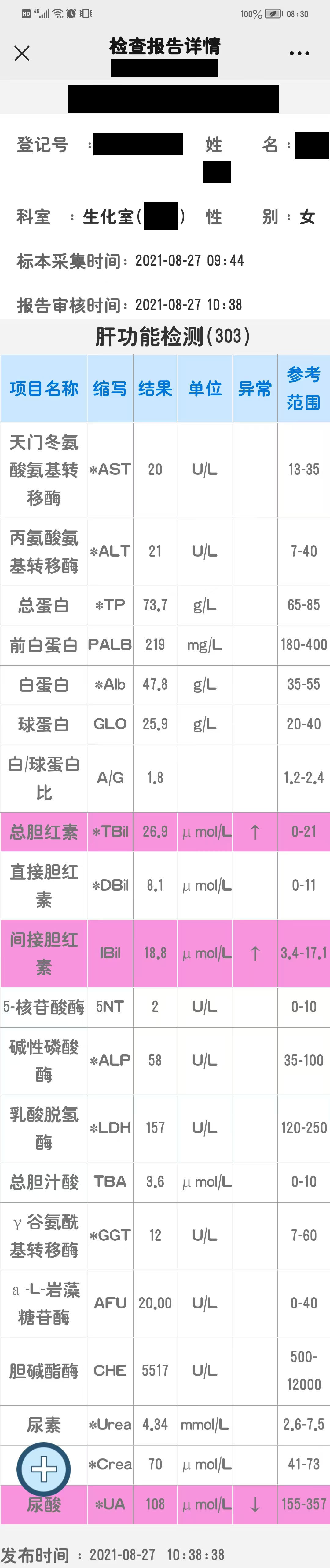

Supplement: Supplementary file 1 [file DataSheet1.zip › original test data/2021.8.27—CMP.jpg]

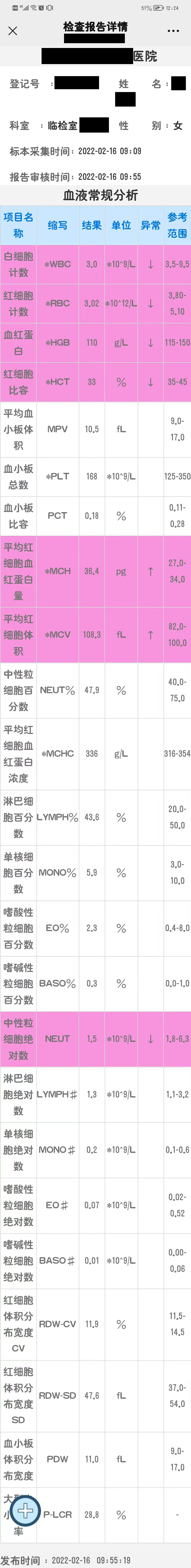

Supplement: Supplementary file 1 [file DataSheet1.zip › original test data/2022.2.16—CBC.jpg]

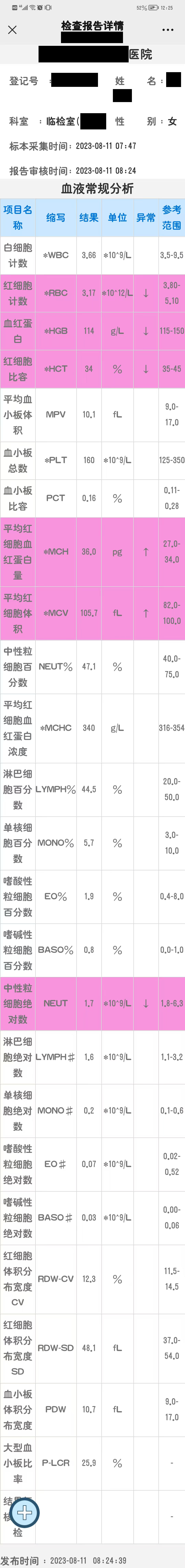

Supplement: Supplementary file 1 [file DataSheet1.zip › original test data/2023.8.11—CBC.jpg]

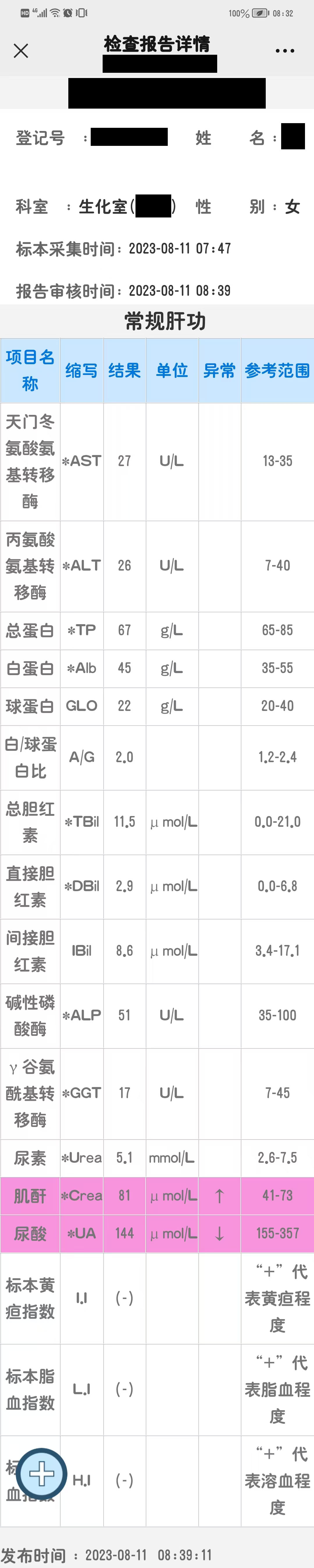

Supplement: Supplementary file 1 [file DataSheet1.zip › original test data/2023.8.11—CMP.jpg]

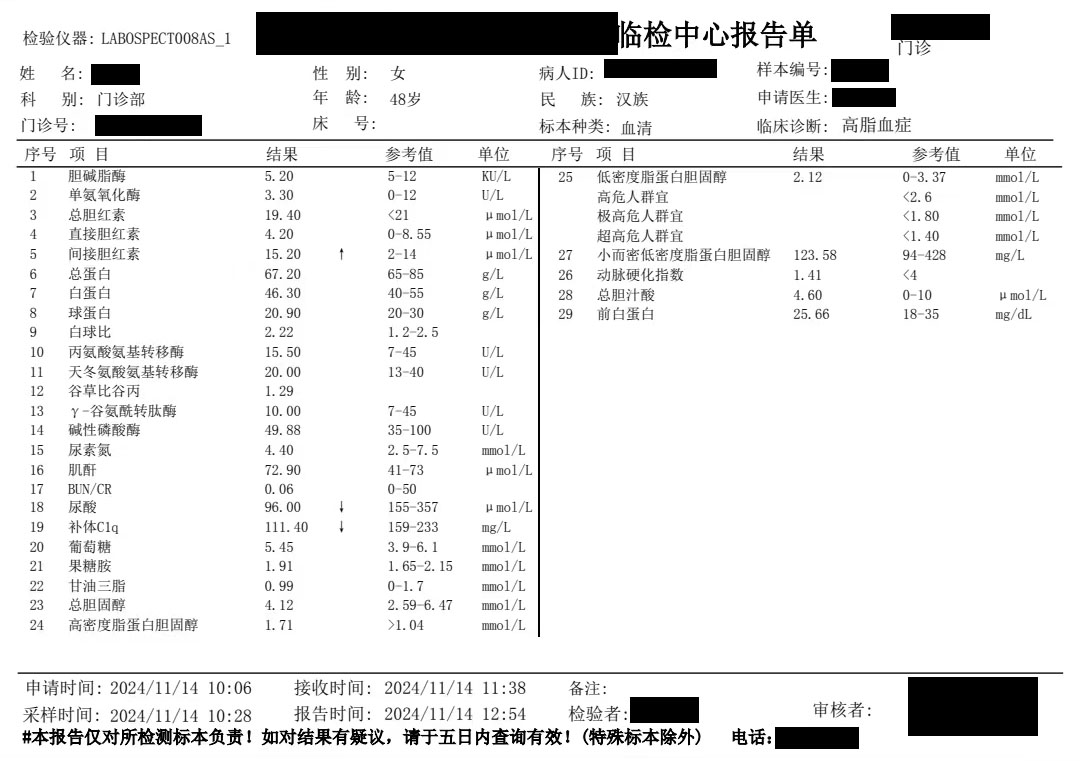

Supplement: Supplementary file 1 [file DataSheet1.zip › original test data/2024.11.14—CMP.jpg]

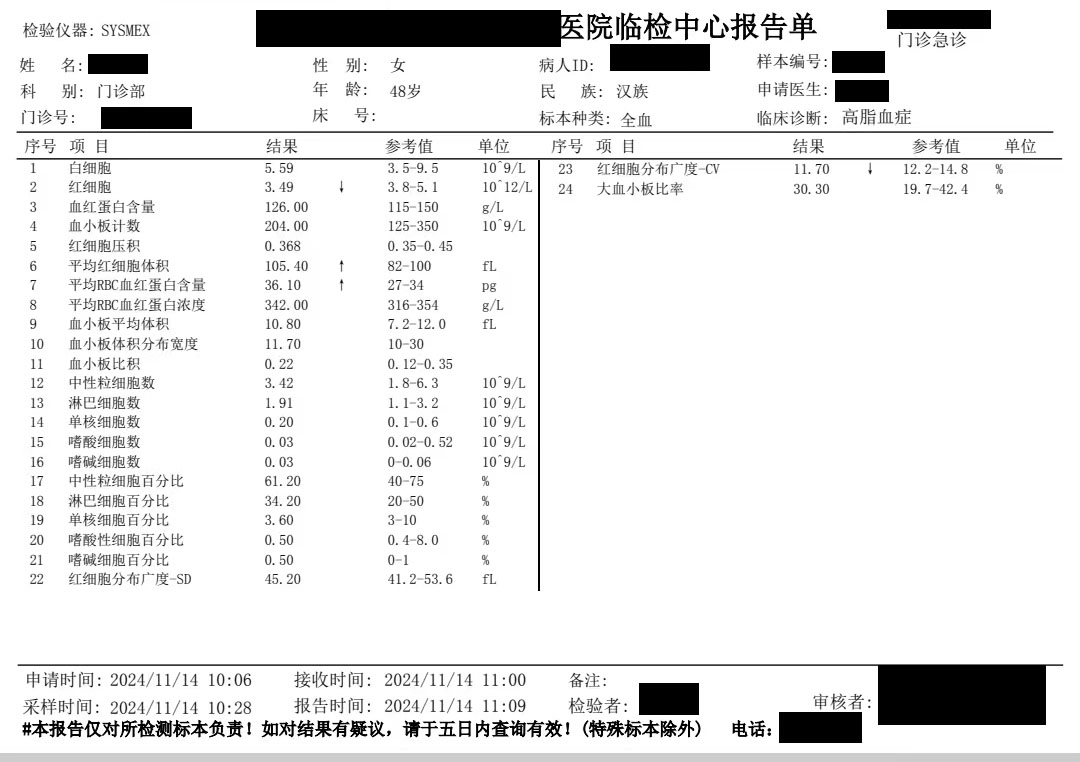

Supplement: Supplementary file 1 [file DataSheet1.zip › original test data/2024.12.14—CBC.jpg]

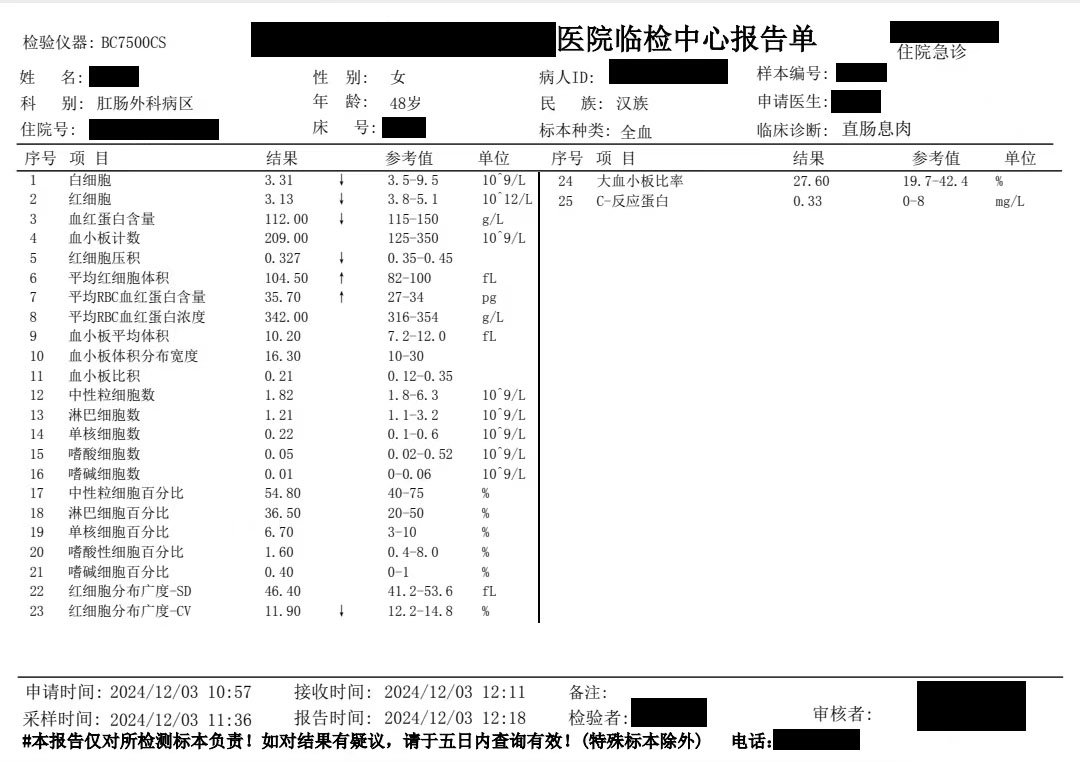

Supplement: Supplementary file 1 [file DataSheet1.zip › original test data/2024.12.3—CBC.jpg]

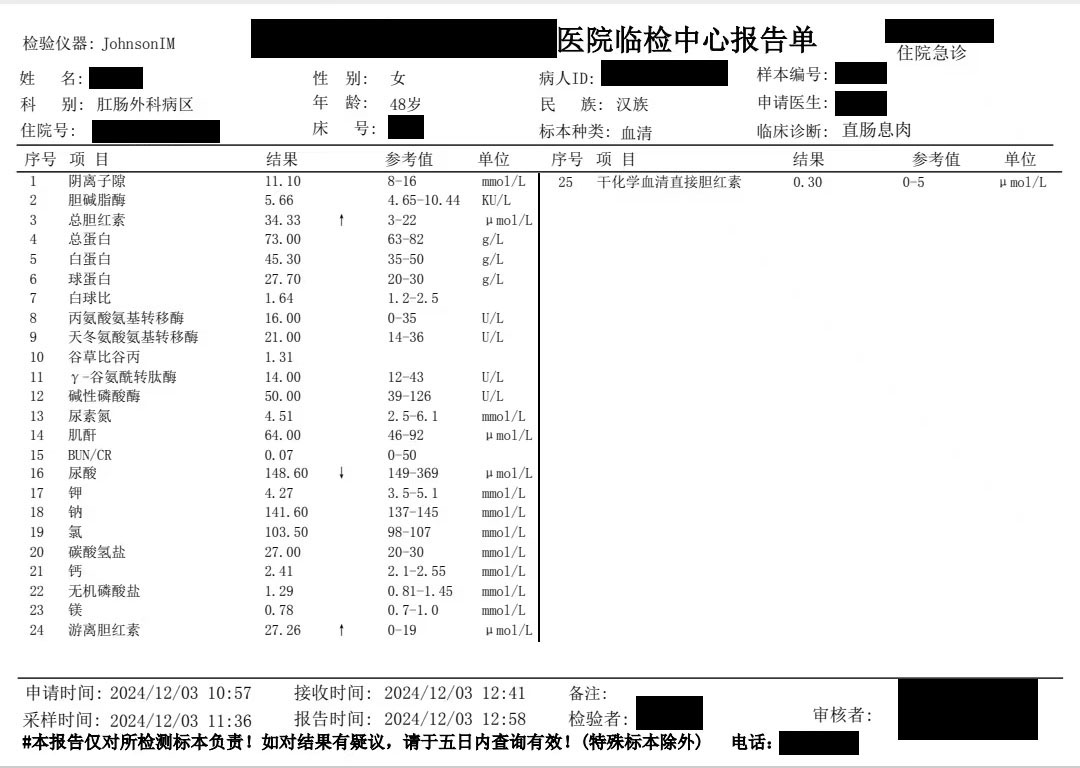

Supplement: Supplementary file 1 [file DataSheet1.zip › original test data/2024.12.3—CMP.jpg]

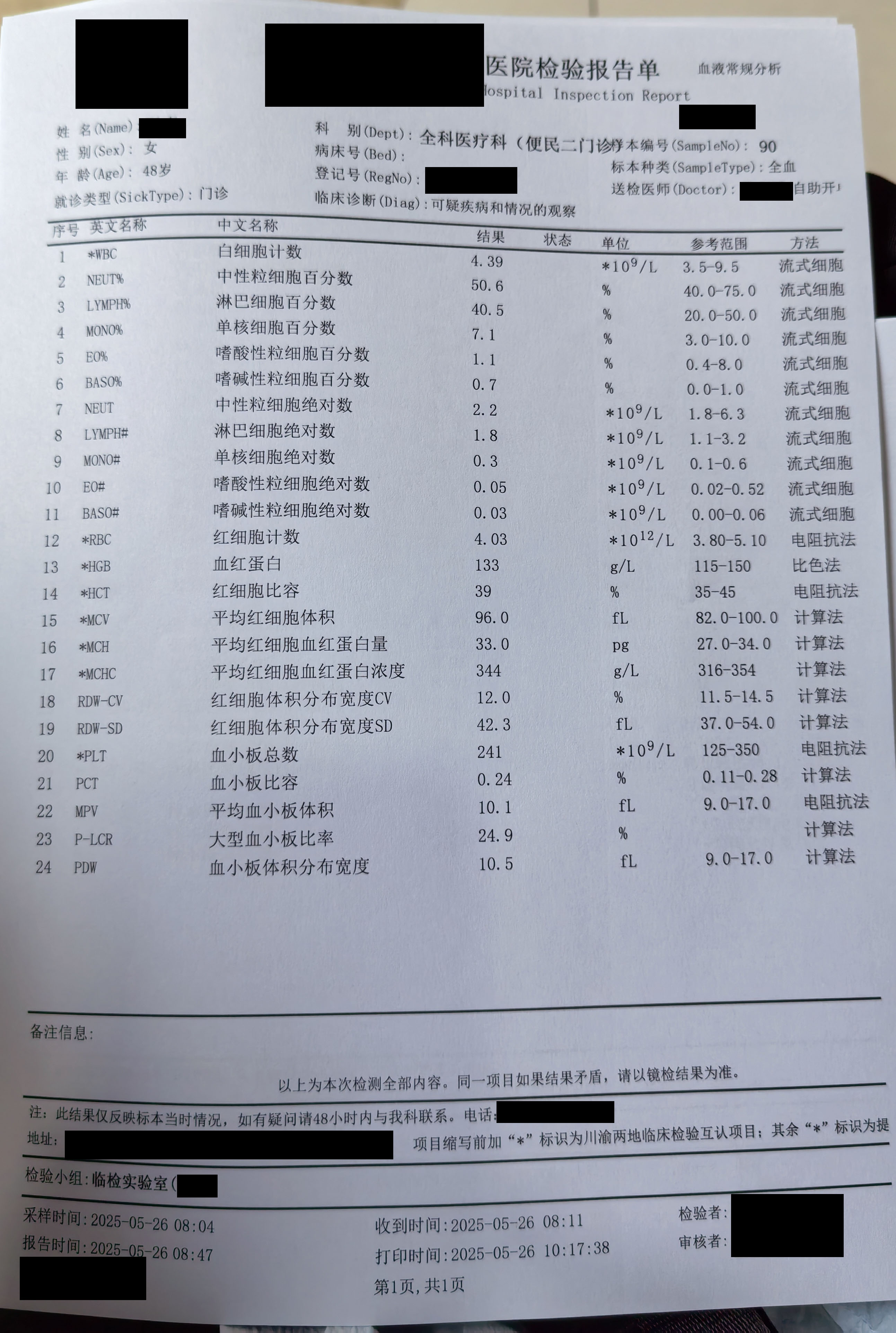

Supplement: Supplementary file 1 [file DataSheet1.zip › original test data/2025.5.26—CBC.jpg]

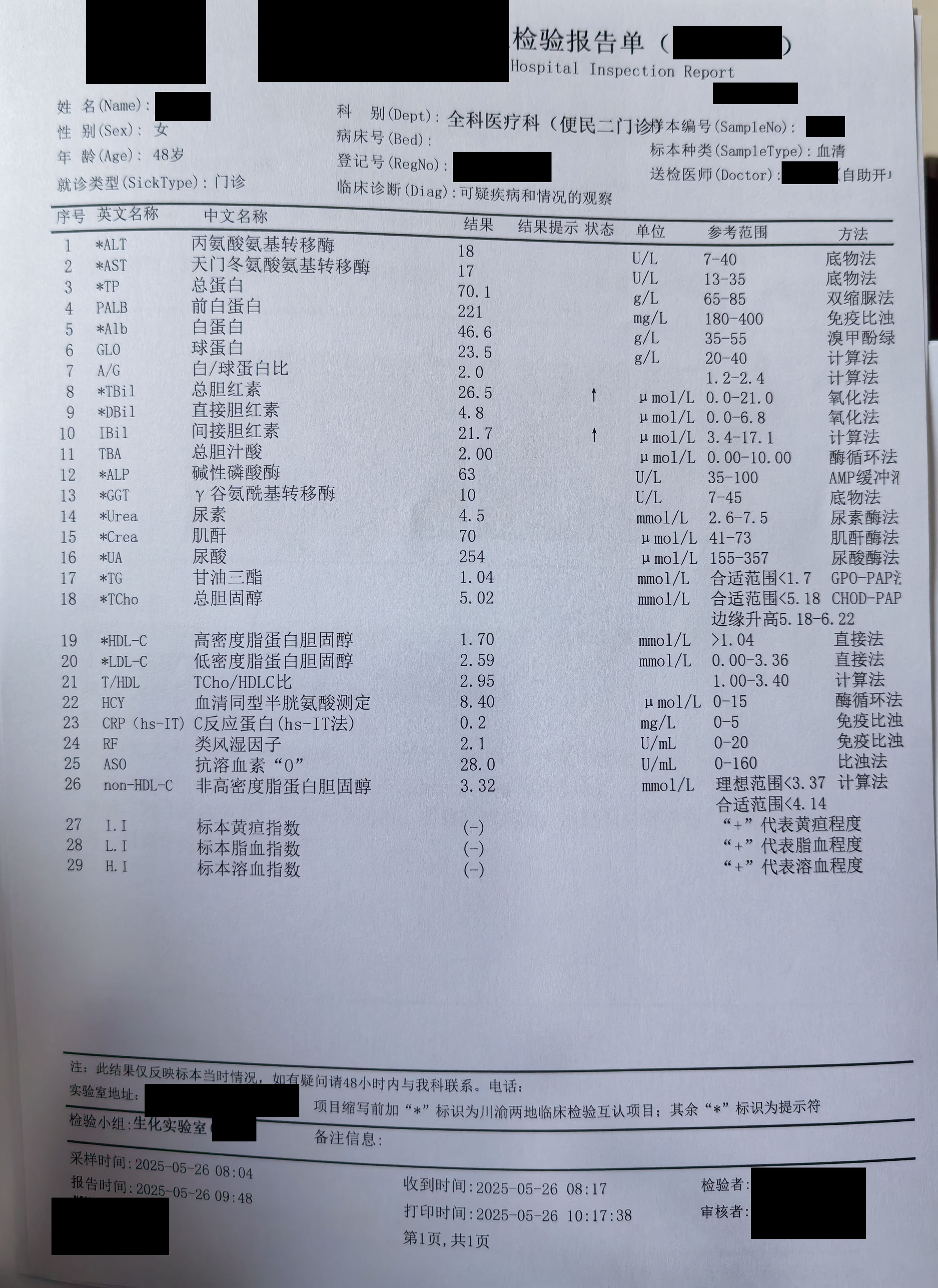

Supplement: Supplementary file 1 [file DataSheet1.zip › original test data/2025.5.26—CMP.jpg]
